# A Y-incision to enlarge the aortic root for aortic valve stenosis with anomalous aortic origin of the right coronary artery

**DOI:** 10.1186/s13019-024-02518-z

**Published:** 2024-02-04

**Authors:** Keisuke Yoshida, Yujiro Miura, Yukiko Fukunaga, Atsuyuki Mitsuishi

**Affiliations:** 1 Department of Cardiovascular Surgery, Kochi Medical School Hospital, 185-1 Kohasu, Oko-cho, Nankoku, 783-8505 Kochi Japan; 2 Department of Surgery, Kochi Medical School Hospital, Kochi, Japan

**Keywords:** Anomalous aortic origin of the coronary artery, Aortic enlargement, Aortic valve stenosis, Aortic valve replacement, Myocardial ischaemia, Y-incision

## Abstract

**Background:**

Anomalous aortic origin of the coronary artery (AAOCA) is a rare congenital heart disease. Therefore, optimal indications for surgery in patients with severe aortic valve stenosis (AS) complicated by AAOCA remain uncertain.

**Case presentation:**

We report the case of a 57-year-old male patient who underwent aortic valve replacement (AVR) and aortic root enlargement using a Y-incision procedure for severe AS with an anomalous aortic origin of the right coronary artery (AAORCA). Since preoperative single-photon emission computed tomography revealed no ischaemic lesions, an aortic root enlargement with a Y-incision was performed to prevent the potential compression of the prosthetic valve on the AAOCA and prosthesis-patient mismatch.

**Conclusions:**

Preoperative evaluation of the coronary anatomy and myocardial ischaemia using advanced imaging modalities and aortic root enlargement with the Y-incision procedure is an effective strategy for preventing ischaemic complications in cases of severe AS with AAORCA.

## Background

Anomalous aortic origin of the coronary artery (AAOCA) is a rare, congenital cardiac abnormality. Although most patients are clinically asymptomatic, certain anomalies are associated with an increased risk of myocardial ischaemia, infarction, and sudden cardiac death. Aortic valve stenosis (AS) is a common valvular heart disease, and its clinical manifestations, such as chest pain, palpitation, and fatigue, are similar to those of AAOCA. No consensus guidelines have been established to standardise optimal surgical therapy for the combination of AS and AAOCA. Additionally, there have been case reports of ischaemic complications related to aortic valve replacement (AVR) in patients with AAOCA [1]. 

Here, we report a novel case of the successful prevention of ischaemic complications by performing AVR and aortic root enlargement with a Y-incision [2] in a patient with severe AS and an anomalous aortic origin of the right coronary artery (AAORCA).

## Case presentation

**Fig. 1:**
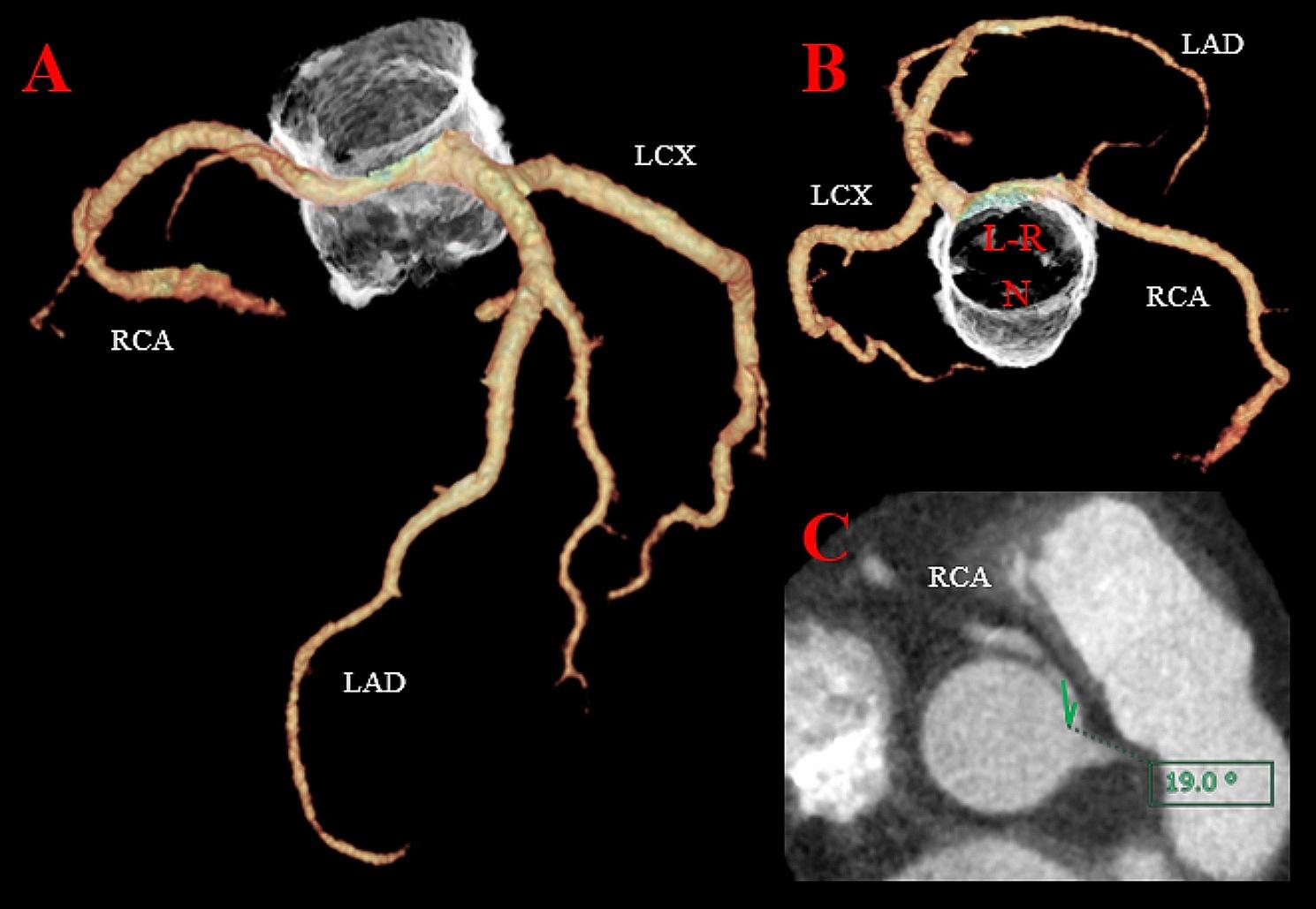
Preoperative cardiac computed tomography. (**A**) The right coronary artery originated from the left coronary ostium. (**B**) Surgeon’s view of the aortic valve. L-R: the fusion of left and right coronary cusps, N: noncoronary cusp (**C**) a take-off angle of the right coronary artery was 19°

**Fig. 2:**
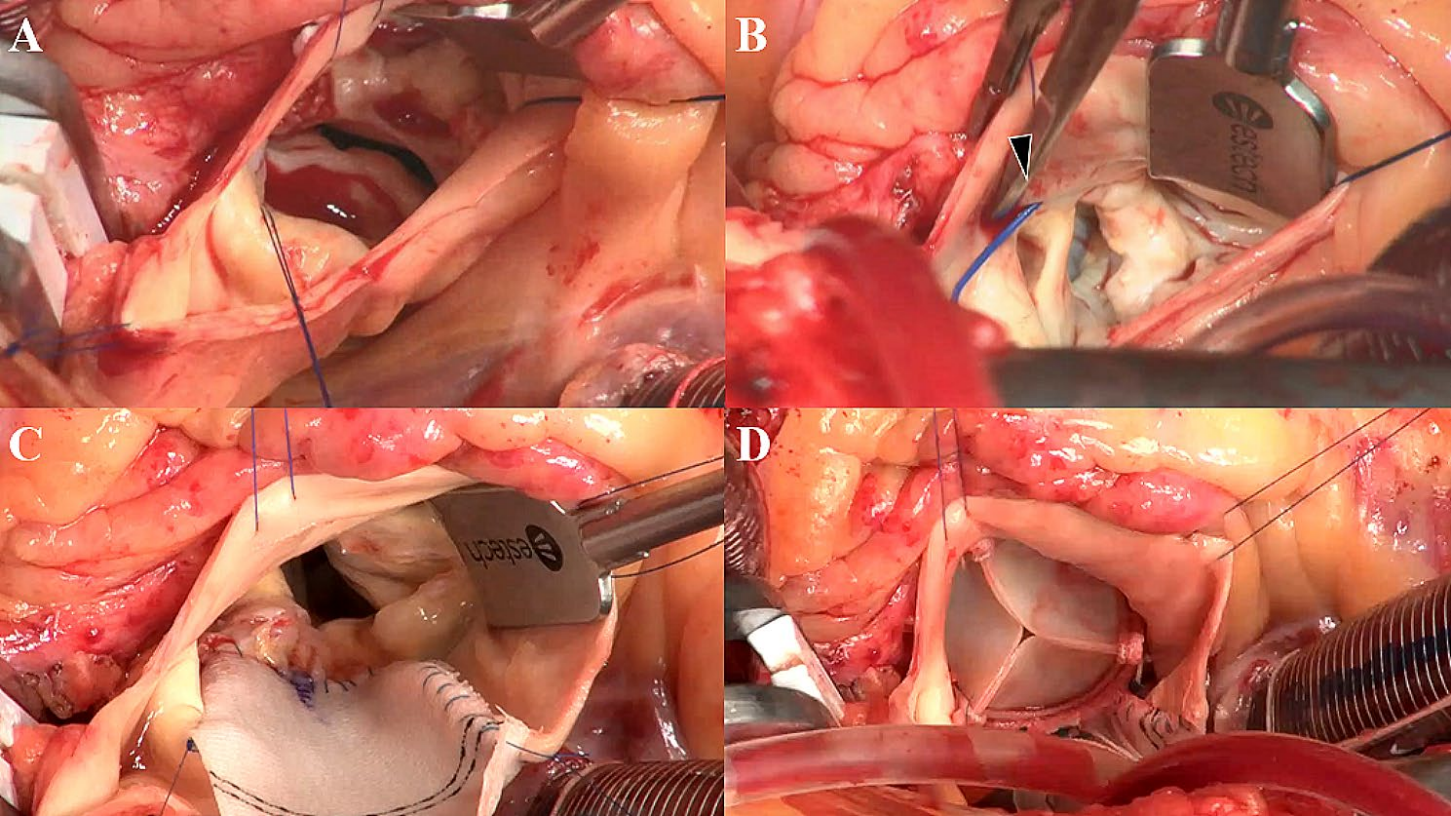
Intraoperative finding. (**A**) Direct visual inspection of the aortic valve confirmed the left-right Sievers 1 phenotype with severe calcification. (**B**) The right coronary artery originated from the left coronary ostium (arrowhead). (**C**) A rectangular patch was sewn to the mitral annulus at the bottom and the aortic annulus on both sides. (**D**) The upsized bioprosthetic valve was seated without compression of the AAORCA

**Fig. 3:**
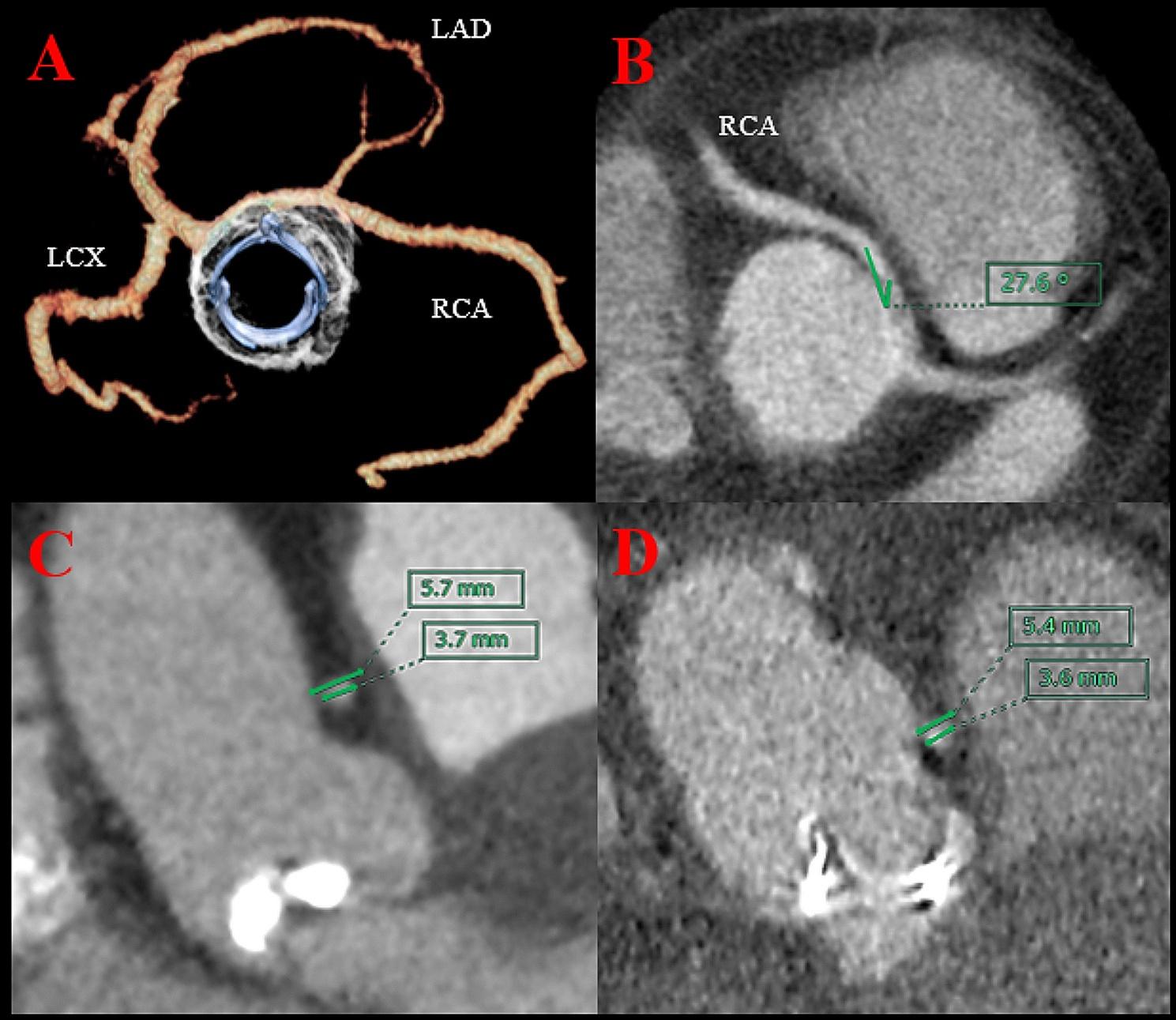
Postoperative cardiac computed tomography. (**A**) After enlargement, the prosthetic valve was placed without compression of the AAORCA. (**B**) A take-off angle of the right coronary artery increased from 19° to 28°. (**C**) The preoperative diameter of the most narrowed point of the interarterial segment and the right coronary artery (RCA) were 5.7 and 3.7 mm, respectively. (**D**) The postoperative diameter of the interarterial segment and the RCA were 5.4 and 3.6 mm, respectively, and not significantly smaller

A 57-year-old male patient who weighed 114 kg, with a height and body surface area (BSA) of 5’6” (170 cm) and 2.19 m^2^, respectively, presented with chest pain and shortness of breath, and was categorised to be under New York Heart Association (NYHA) class III. The patient was referred to our institution for AS evaluation. On admission, the patient’s blood pressure and heart rate were 152/85 mmHg and 71 beats per minute with a regular rhythm, respectively. Transthoracic echocardiography revealed a stenotic bicuspid aortic valve with a calculated valve area of 1.09 m^2^ and a mean gradient of 50 mmHg. The left ventricular ejection fraction was 54% without local asynergy. Coronary angiography exhibited a right coronary artery arising from the left coronary sinus without significant stenosis. Cardiac computed tomography (CCT) revealed that the aortic valve annulus, sinus of Valsalva, and sinotubular junction (STJ) were 24, 31, and 29 mm, respectively. The AAORCA originated from the left coronary sinus at a take-off angle of 19°, which is a known risk factor for myocardial ischaemia (Fig. 1). Although the patient experienced chest pain during the adenosine triphosphate injection, single-photon emission computed tomography (SPECT) revealed no ischaemic lesions. Since the possibility of ischaemic complications was low, we performed AVR without revascularisation. The patient underwent a median sternotomy. The bicuspid aortic valve was classified as Sievers type 1a, characterised by a fusion of the right and left coronary cusps with an asymmetric commissural orientation and the right coronary artery originating from the left coronary ostium. Subsequently, the annular stitches were placed in a non-everting mattress fashion with pledgets after the excision of the leaflets and annular debridement of the calcium. Based on the patient’s preferences, we used a biological prosthesis. A 23-mm prosthetic sizer passed through the annulus; however, mild resistance was noted. Therefore, we enlarged the aortic annulus using a Y-incision for AVR since a less than 23-mm prosthetic valve was insufficient for the BSA, and an oversized prosthetic valve could compress the right coronary artery. After root enlargement using a rectangular Hemashield Dacron patch (Boston Scientific Corp, Natick, Mass, USA), we placed a 25-mm Inspiris RESILIA biologic prosthesis (Edwards Lifesciences, Irvine, California, USA) (Fig. 2). The postoperative aortic valve gradient was 6 mmHg, and the aortic root and STJ diameter increased; the take-off angle of the right coronary artery increased from 19° to 28° without dynamic compression of the interarterial segment (Fig. 3). Finally, the patient was discharged without complications. During the 6-month follow-up, the patient’s weight decreased from 114 to 95 kg with a BSA of 2.06 and NYHA class I.

## Discussion

The symptoms of AAOCA and AS include fatigue, shortness of breath, wheezing, palpitations, angina, and sudden death. Although the 2018 American College of Cardiology/American Heart Association [3] and 2020 European Society of Cardiology [4] guidelines suggested indications for surgery in patients with AAOCA alone, no consensus exists on the optimal surgical therapy for patients with AAOCA and those who require AVR. Therefore, preoperative evaluation of the coronary artery anatomy and myocardial ischaemia is required to determine the surgical decision-making for preventing ischaemic complications. Various diagnostic techniques may be used to investigate the coronary anatomy and assess the presence of high-risk features. Currently, CCT is considered the gold standard, and provocative tests can assess inducible myocardial ischaemia in uncertain cases [5]. In our case, ^99m^Tc-tetrofosmin SPECT was a useful modality for assessing the cause of angina and demonstrated that AAORCA was unassociated with ischaemic symptoms.

Patrick [1] reported compression of the AAOCA with a prosthetic valve in the aortopulmonary continuity after AVR, suggesting under-sizing of the prosthetic valve to prevent ischaemic complications secondary to AAOCA impingement. Here, we performed aortic root enlargement to prevent prosthesis-patient mismatch and compression of the AAORCA and prepare patients for future valve-in-valve transcatheter AVR. The Y-incision procedure is simpler and safer than the Manouguian and Konno procedures and more effective for size-up than the Nicks procedure [2]. Conventionally, the Y-incision procedure is extended in a “Y” fashion, undermining the left-noncoronary annulus to their respective nadir and enlarged root using the patch without significant distortion of the left coronary artery. However, this procedure may modify the left coronary sinus configuration in patients with AAORCA, resulting in a factual rightward shift of the coronary ostia and distortion of the right coronary artery. Therefore, we recommend minimising the left coronary sinus undermining. Moreover, further validation and clinical practice are required to establish the usefulness of the Y-incision for AVR in cases of AAORCA.

## Conclusions

Patients with AAOCA and AS require an evaluation of the coronary anatomy and myocardial ischaemia for surgical indications using advanced imaging modalities. In cases of AS complicated by AAORCA without myocardial ischaemia, aortic root enlargement using the Y-incision procedure is a safe and useful technique that may help prevent fatal ischaemic complications.

## Data Availability

As this paper is a case report, all generated or analysed data are included in this article.

## Abbreviations

AAOCA: Anomalous aortic origin of the coronary artery
AS: Aortic valve stenosis
AVR: Aortic valve replacement
BSA: Body surface area
NYHA: New York Heart Association
CCT: Cardiac computed tomography
STJ: Sinotubular junction
AAORCA: Anomalous aortic origin of the right coronary artery
SPECT: Single-photon emission computed tomography
